# Properties of Basalt Fiber Core Rods and Their Application in Composite Cross Arms of a Power Distribution Network

**DOI:** 10.3390/polym14122443

**Published:** 2022-06-16

**Authors:** Yunpeng Liu, Mingjia Zhang, Hechen Liu, Lin Tian, Jie Liu, Chuanfu Fu, Xiaotao Fu

**Affiliations:** 1 State Key Laboratory of Alternate Electrical Power System with Renewable Energy Sources, North China Electric Power University, Yonghua North Street No. 619, Baoding 071003, China; liuyunpeng@ncepu.edu.cn; 2 Hebei Provincial Key Laboratory of Power Transmission Equipment Security Defence, North China Electric Power University, Yonghua North Street No. 619, Baoding 071003, China; 18367683603@163.com; 3 State Grid Hebei Electric Power Company Electric Power Research Institute, Xingan Street No. 200, Yuhua District, Shijiazhuang 050035, China; tianlin908@126.com (L.T.); 18633015914@163.com (J.L.); 4 Key Laboratory of Physical and Chemical Analysis for Electric Power of Hainan Province, Hairuihou Street No. 23, Haikou 570100, China; fuchuanfu@aliyun.com (C.F.); fuxiaotao123abc@163.com (X.F.)

**Keywords:** basalt fiber, composite materials, insulating materials, core rods

## Abstract

As basalt fiber has better mechanical properties and stability than glass fiber, cross arms made of continuous basalt-fiber-reinforced epoxy matrix composites are capable of meeting the mechanical requirements in the event of typhoons and broken lines in coastal areas, mountainous areas and other special areas. In this paper, continuous basalt-fiber-reinforced epoxy matrix composites were used to fabricate the core rods and composite cross arms. The results verified that basalt fiber composite cross arms can meet the strict requirements of transmission lines in terms of quality and reliability. In addition to high electrical insulation performance, the flexural modulus and the flexural strength of basalt fiber core rods are 1.8 and 1.06 times those of glass fiber core rods, respectively. Basalt fiber core rods were found to be much better load-bearing components compared to glass fiber core rods. However, the leakage current and the result of scanning electron microscopy (SEM) analysis reveal that the interface bonding strength between basalt fibers and the matrix resin is weak. A 3D reconstruction of micro-CT indicates that the volume of pores inside basalt fiber core rods accounts for 0.0048% of the total volume, which is greater than the 0.0042% of glass fiber rods. Therefore, improving the interface bond between basalt fibers and the resin can further improve the properties of basalt fiber core rods.

## 1. Introduction

Composite cross arms are widely used in coastal and mountainous areas due to their light weight, high strength, ease of installation and replacement and significant improvement of the lightning protection performance of the lines [1,2]. Composite cross arms are composed of a silicone rubber shed, composite core rod and an end metal attachment. At present, continuous glass-fiber-reinforced epoxy resin matrix composites (GFRPs) are the main materials used in the manufacture of composite cross-arm core rods. However, some studies have pointed out that the mechanical strength loss of glass fiber is relatively large in saline-alkali environments [3]. GFRPs have high creep resistance under constant load [4,5]. The above problems make it impossible for composite cross arms to meet the requirements for application in coastal areas, mountainous areas and other complex environments.

In most studies, resin modification is used to improve the mechanical properties and stability of core rod materials [6]. However, it is also feasible to start with the improvement of fiber materials. Basalt fiber is made of natural ore after melting and spinning at a high temperature. As basalt fiber is environmentally friendly and stable, it has gradually become an important fiber material in industrial production [7,8,9]. Many studies have confirmed that basalt fiber and its composites have better mechanical properties and stability than glass fiber and its composites. The exudation of basalt fiber elements was found to be able to repair the cracks caused by medium corrosion through acid and alkali corrosion tests [10]. The properties of glass fiber and basalt fiber under high temperature and chemical corrosion were compared elsewhere [11], and the results indicated that basalt fiber has higher strength and more stable properties than glass fiber. The teams of Lopresto V and Dorigato found that basalt fiber laminates are significantly superior to glass fiber laminates in terms of compression, bending, impact resistance and cyclic fatigue resistance by comparing the properties of the two types of fiber laminates [12,13]. Moreover, basalt fiber composites have a damage evolution mechanism different from that of their glass fiber counterparts, with far better bearing capacity and longer service life than the latter [14]. Tests involving immersion in seawater and the brine freeze–thaw cycles of glass fiber composites and basalt fiber composites in the same environment have proven that basalt fiber composites have a higher tolerance to a saline–alkali environment [15,16]. These studies show that it is feasible to use basalt fiber instead of glass fiber to manufacture core rods to improve the mechanical properties and the stability of composite cross arms.

In the present work, the monofilament strength and the interface bonding strength of continuous basalt fiber and continuous glass fiber are compared, and composite cross arms are prepared with the two types of fibers. Dye penetrant tests, core rod hydrothermal tests, core rod-fitting tensile tests, core rod-bending failure tests and internal and external insulation performance tests of composite cross arms are conducted to characterize the internal pores and fiber surface micromorphology of the two types of core rods. The present work is aimed at providing an experimental basis for the application and future improvement of basalt fiber composite cross arms.

## 2. Materials and Methods

### 2.1. Materials

The selected continuous basalt fiber yarn (Sichuan Qianyi, Huaying, China) is 9600 tex, and the continuous glass fiber (Zhejiang Jushi, Jiaxing, China) is 9600 tex. The matrix resin used in the pultrusion process is composed of epoxy resin, curing agent, accelerator and internal release agent in a ratio of 100:75:0.3:10, of which the epoxy resin component (industrial pure, Zhejiang Polimu, Quzhou, China) is diglycidyl ether of bisphenol-A (DGEBA), the curing agent (industrial pure, Zhejiang Polimu, Quzhou, China) is methylhexahydrophthalic anhydride (MHHPA), the accelerator (purity: 95%, Shanghai Macklin, Shanghai, China) is 2,4,6-tris (dimethyl aminomethyl) phenol (DMP-30), and the main component of the internal release agent (commercially pure, Shanghai Macklin, Shanghai, China) is simethicone. The raw material for the sheds is vulcanized silicone rubber (Zhejiang Huabao, Quzhou, China).

### 2.2. Fabrication of Core Rods and Composite Cross Arms

**Figure 1:**
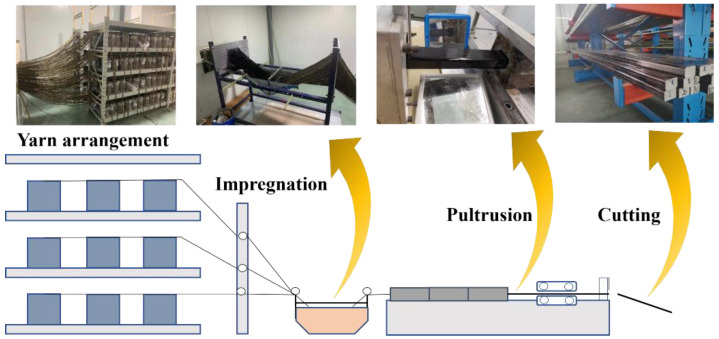
Core rods preparation process.

The process of core rod fabrication is shown in Figure 1. The continuous fiber yarn on the creel was guided and fed into the resin tank to be impregnated with matrix resin. Then, the impregnated yarn was sent to a mold with cross-sectional dimensions of 34 mm × 54 mm for curing. During the whole pultrusion process, the relative humidity of the resin tank should be lower than 40%, the temperature was maintained at 25 °C, the pultrusion rate was 1 m/h and the temperatures of the mold at the three stages were about 120 °C, 150 °C and 150 °C, respectively. The pultruded continuous fiber composite was then cut into lengths of 1100 mm each.

**Figure 2:**
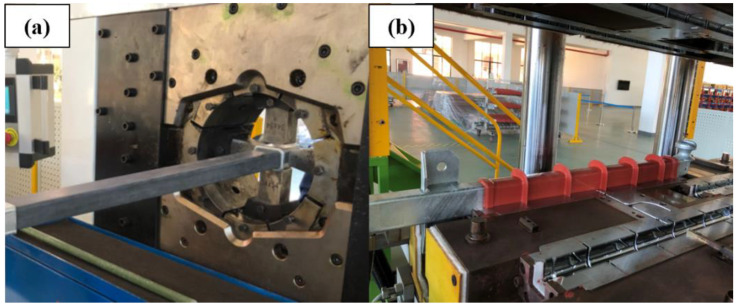
Fitting crimping (**a**) and silicone rubber shed injection (**b**).

The process of obtaining the glass fiber core rods and the basalt fiber core rods by fitting crimping and shed casting is illustrated in Figure 2. The core rods required for the tests were fabricated by crimping the ends of the cut continuous fiber composite with fittings. The coupling agent, ethanol solution, was evenly applied to the surface of the core rods, which were then placed in an oven at 80 °C until the surface became dry. Then, the core rods were placed in the shed injection machine. After the mold was closed, the molten silicone rubber was injected into the mold, and the temperature was increased to 180 °C and held for 120 min to achieve vulcanization of the silicone rubber. After the mold was taken out, the edge of the mold seam and the coated part of the fitting were trimmed and fixed to make the composite cross arms required for the tests.

### 2.3. Characterization

#### 2.3.1. Fiber Properties

The tensile strengths of the two types of fibers were measured in accordance with American Society for Testing Materials (ASTM) C1557-03 standard using a fiber strength testing machine (YG001A, Jigao Instrument, Wenzhou, China) with an initial length of 20 mm and an elongation rate of 5 mm/min. The breaking load of a single fiber and the fiber diameter were recorded to calculate the strength of a single fiber. The Weibull distribution model was used for the statistics of at least 15 sets of significant data.

**Figure 3:**
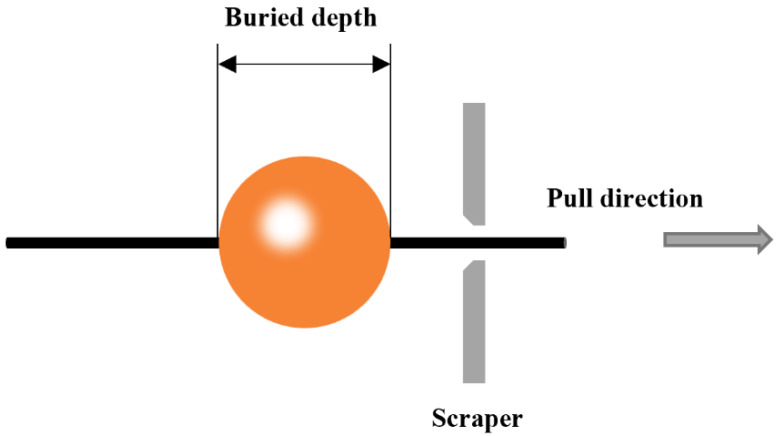
Experimental schematic of the single-microsphere debonding test.

As shown in Figure 3, the debonding method of single-fiber microspheres was used to characterize the difference in the binding ability between the two types of fibers and the resin [17]. The resin was stuck to the fibers and was heated to form cured resin microspheres. The test was carried out with a microsphere debonding testing machine (YG-163, Jigao Instrument, Wenzhou, China). The fibers were passed through the scraper at a speed of 5 mm/min, and the resin microspheres were scraped off by the scraper. By recording the embedding depth of the resin microspheres and the load values when the resin microspheres come off from the fiber, the interface bonding strength between the two types of fibers and the resin can be calculated. The Weibull distribution model was used for the statistics of at least 15 sets of significant data.

#### 2.3.2. Interface Characteristics

To verify the proper bonding of the sheath–mandrel interface and the fiber–resin interface in the core rods, the dye penetrant method was used to test the full-section composite cross-arm samples. The composite cross arm was cut along the direction perpendicular to the fibers to prepare samples with a thickness of 10 mm using grinding wheel cutting. The test process is described as follows: the container was covered with steel balls (each with a diameter of 1 mm). After the sample was placed on the glass balls, an appropriate amount of 1% (by weight) magenta-ethanol solution was poured into the container, with the liquid level 1 mm above the top of the balls. The specimen was allowed to remain still for 15 min to observe whether solvent exudation occurred on the upper surface of the sample.

The hydrothermal test was conducted on the core rod material to compare the interface bonding between the fiber and the resin in the basalt fiber core and the glass fiber core by the magnitudes of the leakage current before and after hydrothermal action. The core rod was cut along the direction perpendicular to the fibers to fabricate samples with a thickness of 30 mm using grinding wheel cutting. The test procedure was carried out as follows: a core rod sample was put into a 0.1% (by weight) sodium chloride solution and boiled for 100 h. Then, the sample was taken out, the surface was wiped, a rated voltage of 12 kV was applied and the leakage current (r.m.s) was recorded using a digital multimeter (RIGIO DM3068).

#### 2.3.3. Mechanical Properties

Considering that the wrapping of the sheath layer could cause measurement error in subsequent mechanical testing, core rods without the sheath were used for mechanical property testing of the composite cross arms. The core rod was 1100 mm long, the dimensions of the cross-section were 34 mm × 54 mm (height × width).

**Figure 4:**
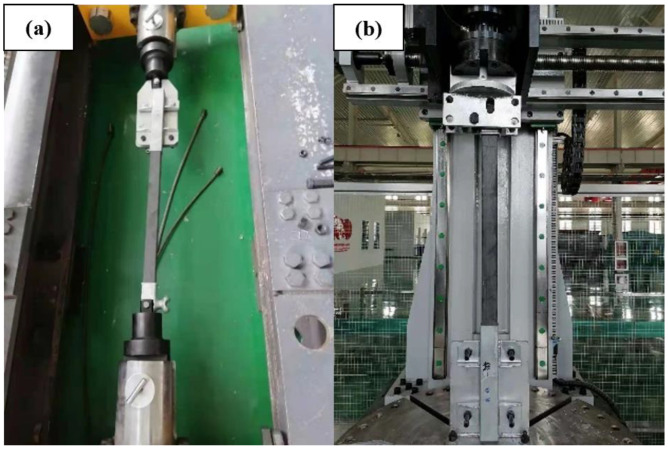
Tensile test (**a**) and bending test (**b**).

The bonding between the core rod and the end metal attachment affects the capacity of the composite cross arms to bear mechanical loads. A tensile load test was used to verify whether changing the core rod material would affect the matching of the core rod and the end metal attachment and whether it can meet the requirements for practical applications. The test equipment was a horizontal tensile testing machine (provided by State Grid Hebei Electric Power Research Institute). The test process is illustrated in Figure 4a. After the test started, a load of 10 kN was quickly and evenly applied and maintained for 90 s, and we observed whether the core rod was pulled out from the end attachment.

To ascertain the difference between basalt fiber core rods and glass fiber core rods in terms of resisting the bending load, bending failure tests were conducted for the two types of core rods. The test equipment is a bending testing machine (provided by State Grid Hebei Electric Power Research Institute). The test process is shown in Figure 4b. The core rod sample was installed on the bending testing machine, and the direction of application of the bending load was perpendicular to the axis direction of the core rod. Then, the load was applied evenly until the core rod failed, and the values of the end displacement and applied load were recorded.

#### 2.3.4. Insulation Properties

The internal insulation strength of composite cross-arm core rods was characterized by the breakdown field strength of the core rods, and the values were measured using a test circuit according to the International Electrotechnical Commission (IEC) 60 243-1 standard. The samples were 1 ± 0.1 mm thick and made by cutting the core rod perpendicular to the fiber direction. The Weibull distribution model was used for the statistics of at least 15 sets of significant data.

Because the external insulation strength mainly depends on the quality of the sheds, the surface flashover test of positive and negative lightning strikes was conducted only on the prepared basalt fiber composite cross arms to obtain their external insulation properties. The sample was 1100 mm long, with a dry arcing distance of 650 mm. The altitude of the test site was 20 m, and the applied lightning impulse voltage waveform was a double-exponential wave of ±1.2/50 μs. The lightning strike surface flashover test was carried out using the Bruceton method to determine the U_50%_ of the basalt fiber composite cross arms based on the mean statistics. Taking the measured U_50%_ voltage as a reference, the test voltage was increased seven times in increments of 10%, the waveform of each flashover was recorded and the positive and negative polarity volt-second characteristic curves of basalt fiber composite cross arms were statistically obtained. During the test, the flashover voltage and meteorological conditions were recorded to correct the test data.

#### 2.3.5. Microtopographic Characterization

The cross section of the core rod was observed after bending failure under high vacuum conditions at an activation voltage of 10 kV with a Nova Nano-450 scanning electron microscope (SEM). The sample was cut from the cracked part of the core rod after the bending test. Considering the high insulation performance of the sample, it was placed on an aluminum holder for sputter coating before observation.

**Figure 5:**
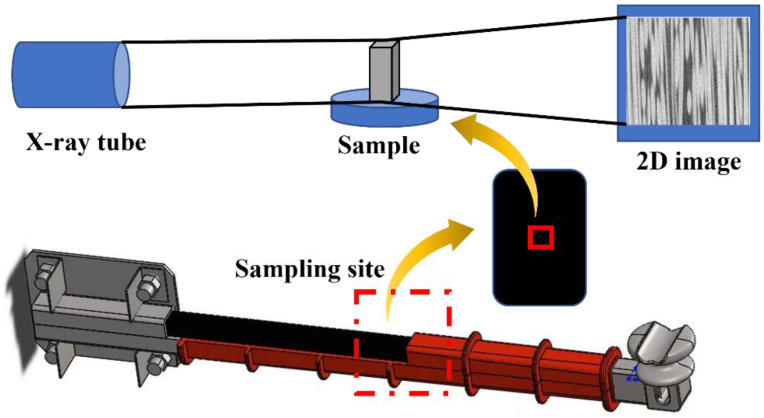
Main stages in X-ray micro-CT.

As shown in Figure 5, a sample measuring 0.4 mm × 0.4 mm × 0.8 mm was drilled on the center of the core rod for observations, and the two types of materials were scanned with a nanoVoxel-3000 high-resolution computed tomography system (CT, Sanying Precision Instruments Co., Ltd., Tianjin, China) with a voxel size of 0.0475 μm. Then, three-dimensional (3D) structural modeling was performed for the scanning results to determine the difference in pore size and pore distribution between the two types of materials [18].

## 3. Results and Discussion

### 3.1. Fiber Properties

**Figure 6:**
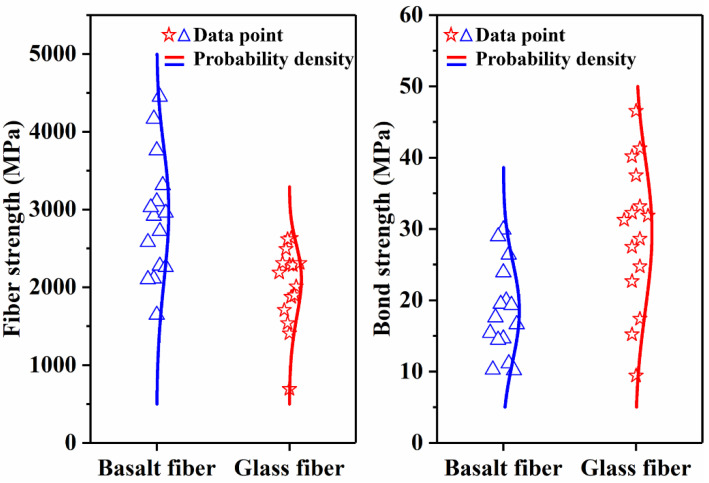
Fiber strength and interface strength comparison.

The fiber strength and the bonding strength of basalt fiber and glass fiber used to prepare the core rods were measured, and the results are shown in Figure 6. The strength at the probability of 63.2% was taken as the failure strength of the fiber and the fiber interface. The monofilament strength of basalt fiber reaches 3186 MPa, which is about 1.45 times that of glass fiber monofilament (the monofilament strength of glass fiber is 2197 MPa). However, the bonding strength between the basalt fiber and the matrix resin is only 20.7 MPa, which is much lower than the 32.6 MPa of glass fiber. These test results show that basalt fiber has better mechanical properties than glass fiber, but the macroscopic properties of the core rods prepared from it may be affected by the weak interface bonding force. The reason for the weak interface bonding is that the basalt fiber has different surface properties. At present, most basalt fiber impregnating compounds use glass fiber impregnating compounds, which cannot improve the properties of such basalt fibers [19]. Therefore, the formulation of impregnating compounds for basalt fiber needs to be improved. In the present work, the core rods were prepared based on the current state of the art of basalt fiber to compare and identify the differences between basalt fiber core rods and glass fiber core rods.

### 3.2. Interface Properties

**Figure 7:**
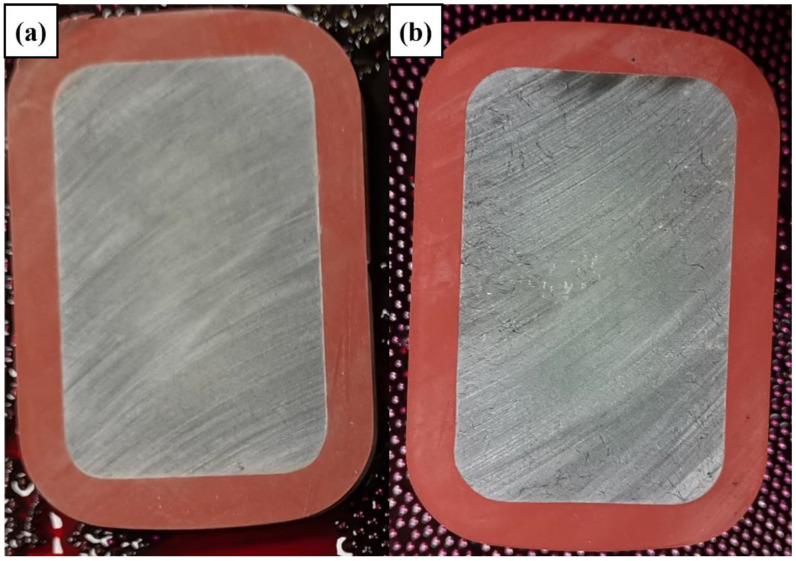
Dye penetration test results for basalt fiber cross arm sample (**a**) and glass fiber cross arm sample (**b**).

The dye penetrant method relies on the capillary action of the test solution to determine whether the sheath of the core rod and the fiber resin inside the core rods have defects throughout the upper and lower parts of the interface [20]. The prepared samples of basalt fiber composite cross arms and glass fiber composite cross arms were tested, and the results are shown in Figure 7.

No dyeing solution was drawn onto the surface by capillary action in any part of the sample. In the glass fiber core rods and the basalt fiber core rods, there were no defects throughout the upper and lower parts of the interface, and the silicone rubber shed was well-bonded with the two types of core rods, without pore defects through the upper and lower parts of the interface.

**Figure 8:**
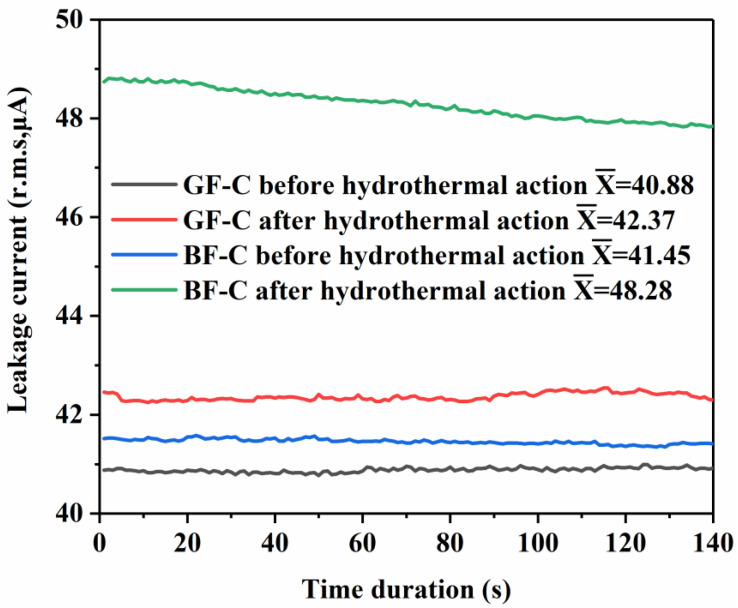
Leakage current amplitude before and after hydrothermal action.

A hydrothermal action test was used to verify the reliability of the fiber–resin interface in the core rods [21]. If there are pore defects or areas with poor interface bonding inside the sample, the leakage current of the sample will change in amplitude under the hydrothermal action, and breakdown will occur in the channel throughout the sample in severe cases. The leakage current amplitudes of the basalt fiber core rods and the glass fiber core rods before and after hydrothermal action are illustrated in Figure 8 (GF-C represents the glass fiber core rods, and BF-C denotes the basalt fiber core rods).

Before the hydrothermal action, the leakage currents of the glass fiber core rod and the basalt fiber core rod were similar. The mean leakage current amplitudes are 40.88 μA and 41.45 μA, respectively, and the amplitude remains stable with the increase in the pressurization time. During the hydrothermal test, the NaCl solution penetrated the internal defects of the core rod, which eventually led to an increase in the leakage current amplitudes of the two types of core rods. The amplitude of the glass fiber core rods increased by 1.49 μA, whereas that of the basalt fiber core rods increased by 6.83 μA. The reason for such a difference is that the interface bonding of basalt fiber is relatively weak. Although the leakage current in the hydrothermal test was weaker than that of the glass fiber core rods, the basalt fiber core rods still meet the standard requirements, which demand that the leakage current of the core rod sample after the hydrothermal action does not exceed 50 μA.

### 3.3. Mechanical Properties

**Figure 9:**
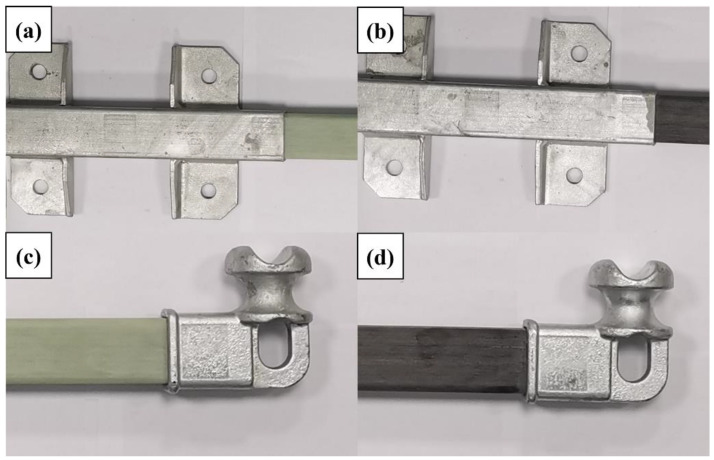
End fitting connection (**a**,**b**) and wire fitting connection (**c**,**d**).

The composite cross-arm core rod and the end metal attachment are the key components for load bearing, so the bonding between them is very important. In the crimping process, the metal fittings are slightly deformed by an external force, and the core rod is only slightly elastically deformed. Tensile testing proves whether the prepared core rod-fitting bond meets the operating requirements. Figure 9 shows photos of the fittings after the tensile load was applied to the two types of core rods. The two types of composite cross arms suffered no failure or falling off of the fittings and were well bonded thereto.

**Figure 10:**
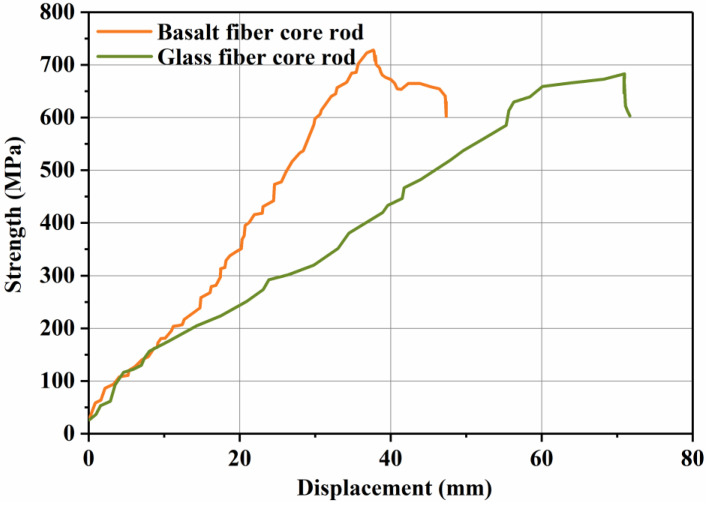
Bending load–displacement curve.

The composite cross arms were fixed laterally on the towers when they were mounted to support the transmission lines. In addition to bearing the dead load and the wind load of the lines and other accessories, they also need to resist the tension from the direction of the wires. Therefore, the ability to resist bending failure is very important for the safe and stable operation of composite cross arms. Meanwhile, the bending strength and modulus are also key parameters for the design of the cross-section size of composite cross arms. Table 1 shows that due to the high mechanical properties of basalt fiber, the flexural resistance of basalt fiber core rods is much greater than that of glass fiber core rods. The flexural modulus and the flexural strength of basalt fiber core rods are 1.8 and 1.6 times those of glass fiber core rods, respectively. The improvement in flexural resistance implies that the creep of basalt fiber composite cross arms under operating conditions is reduced, and a higher flexural modulus enables a smaller cross section to be used to meet the requirements for the deflection of composite cross arms [22]. Figure 10 indicates that there are fluctuations in the failure process of the basalt fiber core rods, whereas the glass fiber core rods cannot bear any load failure. The reason for the above phenomenon is that the glass fiber core rods can better distribute the stress to the fibers during the load bearing process, which will eventually cause the fibers to fail instantaneously when they reach their ultimate bearing capacity. While the basalt fiber core rods are under load, cohesive failure occurs at the resin of the interface layer first, so failure is accompanied by fiber pull-out and other results [23].

### 3.4. Insulation Properties

**Figure 11:**
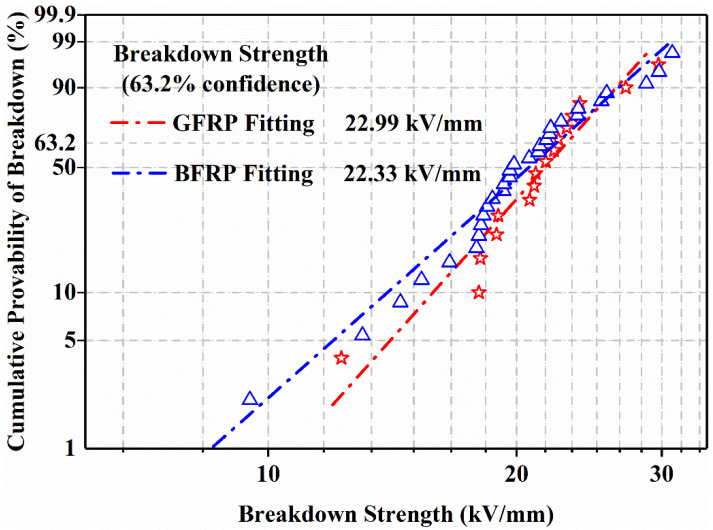
Weibull distribution of the breakdown strength.

In the present work, the Weibull distribution was used to determine the breakdown field strength of glass fiber core rod materials and basalt fiber core rod materials, and a breakdown probability of 63.2% was employed to represent the breakdown field strength of the medium. The test results are shown in Figure 11. There is only a slight difference in the breakdown strength of basalt fiber core rods and glass fiber core rods. When the breakdown field strength exceeds 22 kV/mm, both can provide sufficient insulation strength for composite cross arms.

**Figure 12:**
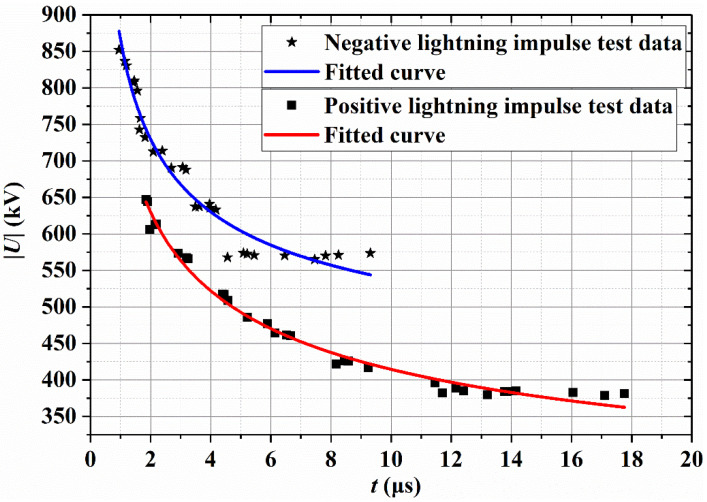
Positive- and negative-polarity volt-second characteristics.

A lightning-strike surface flashover test was conducted for basalt fiber composite cross arms to verify their lightning protection performance. To eliminate the influences of temperature and humidity on the discharge, the g-parameter method was used to correct the temperature to the standard temperature of 20 °C and the humidity to the absolute humidity of 11 g/m^3^. The positive- and negative-polarity U_50%_ values of basalt fiber composite cross arms are shown in Table 2. When the altitude is 0 m, the positive-polarity U_50%_ of composite cross arms with an effective insulation distance of 0.65 m reaches 399.40 kV, and the negative-polarity U_50%_ is 564.22 kV, both meeting the lightning protection requirements for more than 350 kV. Figure 12 shows the positive- and negative-polarity volt-second characteristic curves of basalt fiber composite cross arms; the expression of the positive-polarity volt-second characteristic curve is as shown in Equation (1), and the expression of the negative-polarity volt-second characteristic curve is indicated in Equation (2). The positive and negative volt-second characteristic curves were obtained to facilitate lightning protection analysis when basalt fiber composite cross arms are used.

(1) $$V_{s-t}=209.1\;d+\frac{1031.5\;d}{t^{0.350}}$$

(2) $$V_{s-t}=-541.5\;d\;-\frac{777.2\;d}{t^{0.447}}$$

### 3.5. Microtopography

**Figure 13:**
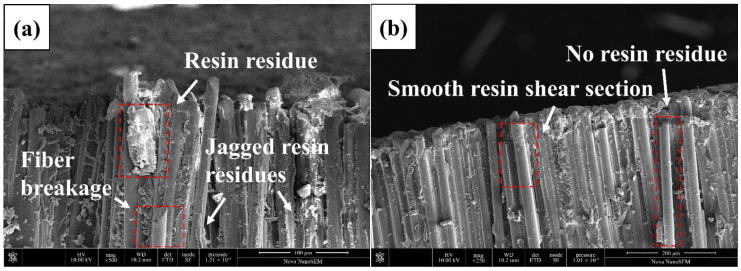
Glass fiber core rod section (**a**) and basalt fiber core rod section (**b**).

To further explore the interface difference between the two types of core rods, the fracture sections of the two types of core rods were observed. Figure 13a shows the SEM for the cross section of the glass fiber core rod, and Figure 13b illustrates the SEM for the cross section of the basalt fiber core rod. There is little residual resin at the cross section of the basalt fiber core rod, whereas there is a considerable amount of residual resin at the cross section of the glass fiber core rod. There are also jagged resin fragments and broken fibers on the surface of the glass fiber, whereas there is no resin residue or a relatively smooth resin section on the surface of the basalt fiber. The reason for the above phenomenon is that the good interface bonding of the glass fiber core rod absorbs a large amount of fracture energy, resulting in resin wrinkles and jagged fragments, giving full play to the fiber strength to cause the fiber to break when bearing any load. However, the interface bonding of basalt fiber is too weak to give full play to the fiber strength, and cohesive failure occurs at the resin of the interface layer when subject to external force, thus forming a smooth resin cross section [24,25].

In order to further determine the interface bonding effect of the two types of core rods, a sample measuring 0.4 mm × 0.4 mm × 0.8 mm was taken from the center of the core rod for microcomputed tomography, and the CT image was visualized in 3D with Avizio^®^ to generate an internal pore image of the sample. The resulting pore image information was processed by the Pore Network Model module to obtain the throat information of the pores inside the sample.

**Figure 14:**
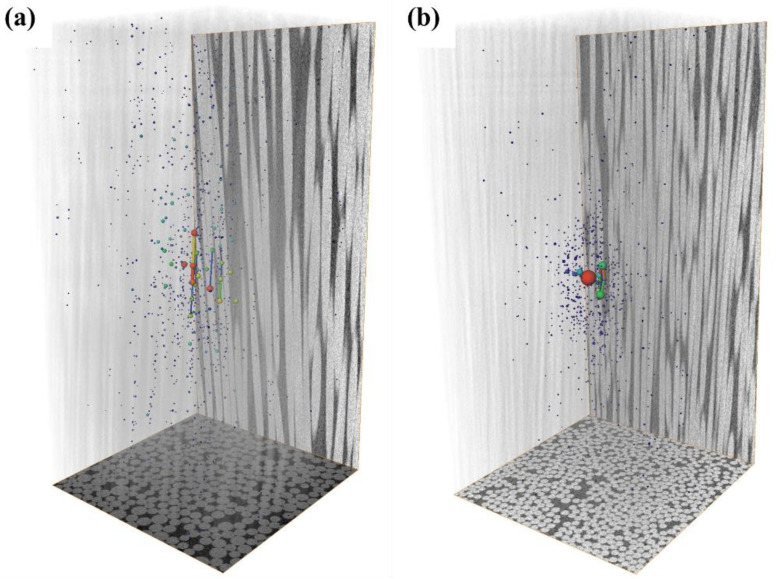
Three-dimensional pores of basalt fiber core rod (**a**) and glass fiber core rod (**b**).

**Figure 15:**
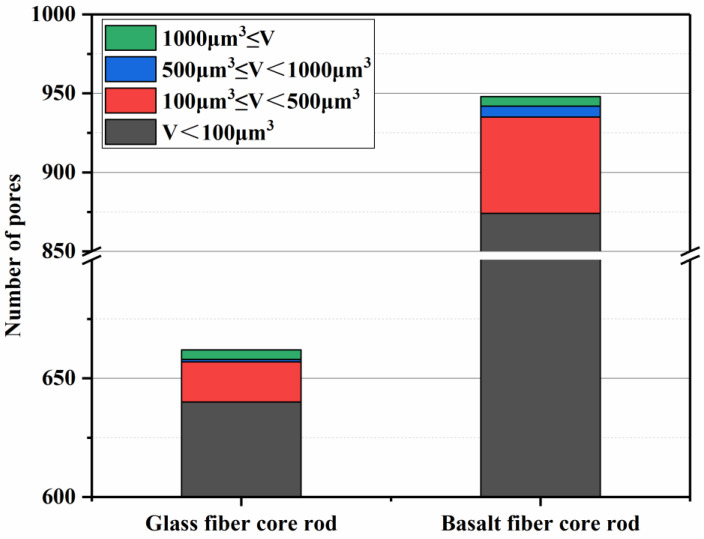
Different volume porosity content in two types of core rods.

Figure 14 shows the internal pores of the glass fiber and the basalt fiber core rod samples. There is a significant difference in pore volume between the two. The internal pore volume of the glass fiber core rod sample accounts for 0.0042% of the total volume, whereas that of the basalt fiber core rod sample accounts for 0.048%. Figure 15 displays the proportion of pores in different volumes inside the two types of samples. Although both have a similar pore volume distribution, with the highest proportion of pores below 100 μm^3^, the glass fiber core rod has far fewer pores than the basalt fiber core rod. The model also shows connected pore throats, with the throat features listed in Table 3. The number of pore throats in basalt fiber is greater than that in glass fiber, and the pore throats of basalt fiber are narrow and long channels, whereas those of glass fiber are shorter and closer to the pore structure. The reason for the above phenomenon is that the impregnation effect of the matrix resin on the basalt fiber is not as good as that on the basalt fiber. Therefore, during the pultrusion process, most of the pore defects of the basalt fiber are caused by the failure of the resin to spread timeously, and most of the pore defects of the glass fiber are caused by the failure to discharge the air bubbles in time, which are compressed to the center.

## 4. Conclusions

Basalt fiber core rods were prepared for composite cross arms, and a penetrant test, hydrothermal test, core rod-fitting tensile test, bending failure test, core rod breakdown field strength test and lightning strike surface flashover test were conducted on the prepared basalt fiber composite cross arms. The differences in leakage current, bending failure performance and breakdown field strength of basalt fiber core rods and traditional glass fiber core rods before and after the hydrothermal action were compared. The reasons for the performance differences were studied by SEM and microcomputed tomography. The following conclusions can be drawn:

1. Due to the good mechanical properties of basalt fiber, basalt fiber core rods are superior to glass fiber core rods in terms of flexural modulus and failure deflection. Basalt fiber core rods are better load-bearing members than glass fiber rods.
2. The breakdown field strength of basalt fiber core rods is only slightly different from that of commonly used glass fiber core rods, so they can be used as a reliable insulating medium for composite cross arms. In addition, the positive- and negative-polarity U_50%_ values of composite cross arms made of basalt fiber core rods are much higher than 350 kV.
3. The prepared basalt fiber composite cross arms can pass the dye penetrant test, hydrothermal test, core rod-fitting tensile test and lightning strike surface flashover test, meeting the strict requirements of transmission lines in terms of quality and reliability of composite cross arms.
4. SEM and the microcomputed tomography show that there are differences in the interface bonding of basalt fiber core rods and glass fiber core rods, with the former being slightly weaker than the latter. Through 3D reconstruction, the number and size of internal pores of the basalt fiber core sample were found to far exceed those of the glass fiber core rod sample.
5. At present, the conventional impregnating compounds for glass fiber are mostly used for basalt fiber processing, which cannot generate satisfactory treatment effects on basalt fiber due to the difference in surface properties. The development of basalt fiber impregnating compounds suitable for the power generation sector can further improve the excellent properties of basalt fiber.

## Figures and Tables

**Table 1:** Comparison of bending parameters between glass fiber core rods and basalt fiber core rods.

	Glass Fiber Core Rod	Basalt Fiber Core Rod
Bending modulus (GPa)	63.29	114.07
Bending strength (MPa)	682.67	727.97
Displacement (mm)	70.96	37.68

**Table 2:** Positive- and negative-polarity U_50%_ of Basalt fiber composite cross arms.

Polarity	Dry Arc Distance (m)	U_50% test_ (kV)	U_50%_ (kV)
+	0.65	380.11	399.40
−	0.65	569.98	564.22

**Table 3:** Pore throat characteristics of basalt fiber core rods and glass fiber core rods.

	Basalt Fiber Core Rod	Glass Fiber Core Rod
Total number of throats	9	6
Average throat area (μm^2^)	23.46	46.27
Average throat radius (μm)	2.57	3.76
Average throat length (μm)	40.51	28.94

## Data Availability

The data presented in this study are available on request from the first authors and corresponding author.
